# Control of inferior vena cava injury during laparoscopic surgery using a double balloon-equipped central venous catheter: proof of concept in a live porcine model

**DOI:** 10.1007/s00464-017-5938-6

**Published:** 2017-11-03

**Authors:** Yukio Iwashita, Hiroki Uchida, Hiroomi Takayama, Michihiro Ichimanda, Katsuya Taniguchi, Hideki Kiguchi, Tetsumi Sakaguchi, Hajime Fujishima, Kunihiro Saga, Kazuhiro Tada, Takao Hara, Kiminori Watanabe, Teijiro Hirashita, Yuichi Endo, Masayuki Ohta, Masafumi Inomata

**Affiliations:** 1 Department of Gastroenterological and Pediatric Surgery, Oita University Faculty of Medicine, 1-1 Hasama-machi, Yufu, Oita 879-5593 Japan; 2 TOGO MEDIKIT Co., LTD., Miyazaki, 883-0062 Japan

**Keywords:** Inferior vena cava injury, Central venous catheter, Laparoscopic liver resection, Massive hemorrhage

## Abstract

**Background:**

Iatrogenic inferior vena cava (IVC) injury is a rare but potentially life-threatening complication during laparoscopic surgery. This experimental study aimed to assess the hemostatic ability of a new device, double balloon-equipped central venous (DB-CV) catheter, for IVC injury.

**Methods:**

The DB-CV catheter comprises a triple-lumen sphincterotome combined with two dilating balloons having a diameter of 25 mm. The experimental procedures were performed in five pigs. The DB-CV catheter was inserted via the right femoral vein. For the IVC occlusion test, correct placement of the balloons was confirmed by indocyanine green fluorescence imaging, and hemodynamic data were recorded. For the IVC injury test, a 3- to 4-mm circumferential incision was created in IVC, and hemostasis was initiated using balloon inflation 5 s after the injury.

**Results:**

Hemodynamic changes were minimal, with a 20 mmHg reduction in the mean arterial pressure because of IVC occlusion. All bleeding from IVC injuries was successfully temporarily stopped by direct balloon compression, with a mean time to hemostasis of 69 s and mean blood loss of 32 ml. Subsequently, the positioning of IVC injuries between two balloons made it possible to suture the injured IVC.

**Conclusions:**

Balloon occlusion using the DB-CV catheter provides a rapid temporal hemostatic effect and can overcome the serious condition of massive hemorrhage from IVC injuries.

The number of laparoscopic liver resections being performed is increasing worldwide [1]. With the developments of electronic hemostatic devices and improvements in surgical techniques, laparoscopy can be adapted to major hepatic resection, including hemi-hepatectomy [2]. Separation of hepatic parenchyma from the inferior vena cava (IVC) is sometimes necessary during major operations. The incidence of iatrogenic IVC injury during laparoscopic surgery reportedly ranges from 0.01 to 1.98% [3–5]. As performing major laparoscopic hepatectomy increases, iatrogenic IVC injuries may become more frequent. Control of bleeding and repair of IVC injury during laparoscopic surgery is more technically demanding than during open laparotomy [2]. Simply compressing with gauze, using a clip, or applying forceps is often demanding because of the inherent movement constraints during a laparoscopic procedure. Therefore, it is highly recommended to convert to an open surgery if IVC injury occurs. However, during emergency laparotomy, several minutes are required to reach the injury site, and further blood loss may result from removal of pneumoperitoneum pressure. Here, we developed a new device, a double balloon-equipped central venous (DB-CV) catheter, to control bleeding immediately after IVC injury. This study aimed to evaluate the effect and technical feasibility of a DB-CV catheter for controlling severe IVC hemorrhage in a live porcine model.

## Materials and methods

### Prototype device

**Fig. 1:**
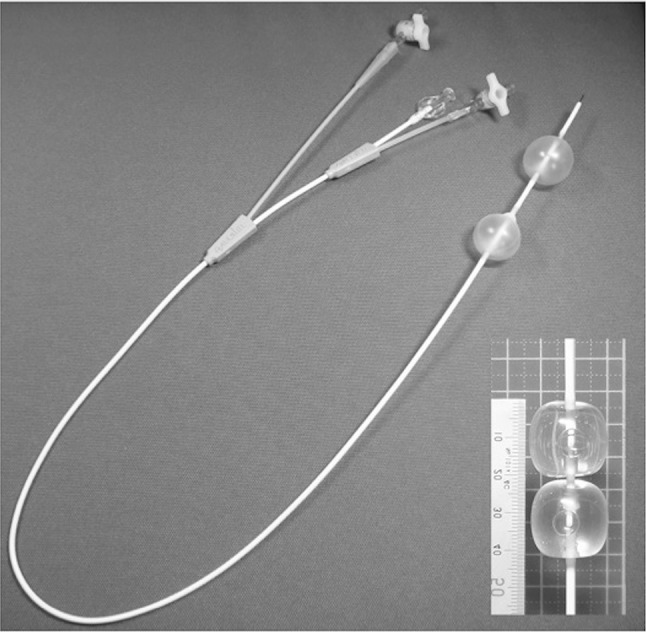
Double balloon-equipped central venous catheter and tip of the dilating balloon

The balloon-equipped CV catheter prototype is 7 Fr in diameter and 70 cm in working length. It comprises a single-lumen CV catheter combined with a double balloon (Fig. 1). The two balloons are located serially, towards the distal end of the CV catheter, with a gap in between, which could be placed at the site of injury, to enable suturing of the tear with a bloodless field enabled by the inflated balloons occluding the IVC proximal and distal to the injury; both are proximal to the catheter lumen for injection and are 25 mm in maximum diameter and 30 mm in length. The balloons can be individually inflated through an additional lumen using a standard inflation device.

### Preparation of animals

**Fig. 2:**
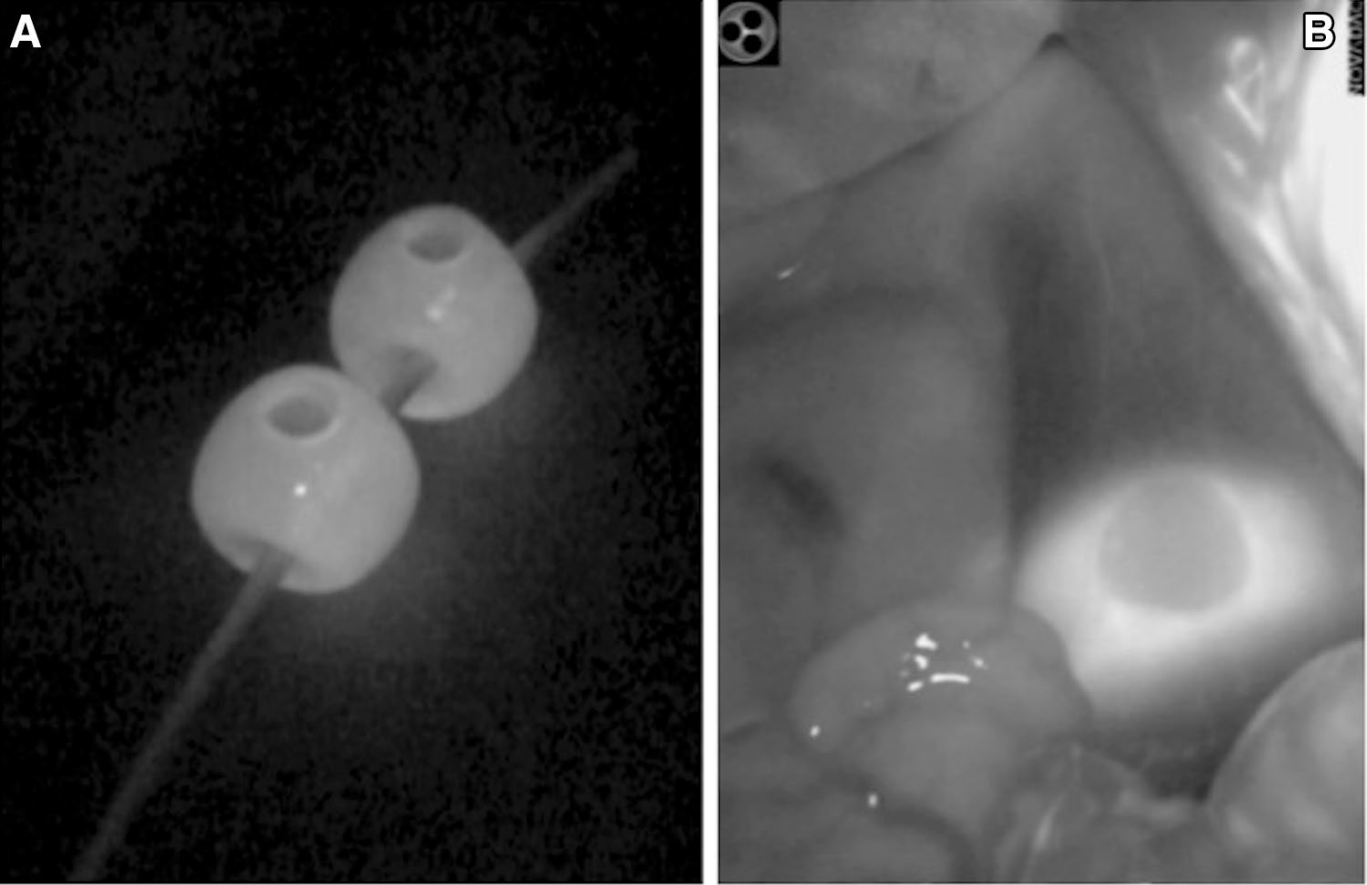
Indocyanine green (ICG) fluorescence imaging. The catheter balloons were inflated with ICG solution (0.025 mg/ml) plus a drop of blood. **A** Before catheter insertion. **B** The intravenous position of the balloon was confirmed using near-infrared fluorescence

The study protocol was approved by the animal research committee at Oita University Faculty of Medicine (Approval No. R020002), and all animals were managed according to the ethical guidelines for animal studies. Five female pigs (Kyudo Company, Japan) weighing 30–40 kg were used in the study. They were deprived of food overnight before the experiments but had free access to water. Under general anesthesia, an arterial line was inserted in the right external jugular artery for continuous arterial pressure monitoring. Electrocardiogram leads were placed and connected to a monitor. CO_2_ pneumoperitoneum was established at 10 mmHg through a Hasson’s trocar. Four or five working ports were placed. IVC was exposed by retracting small bowel and pancreatic glandular tissues. The right femoral vein was then dissected for catheterization with the DB-CV catheter. In this study, we used a laparoscopic indocyanine green fluorescence (ICG) imaging navigation system (Pinpoint; Novadaq Technologies, Canada) to ensure accurate placement of balloons. The balloons of the catheter were inflated with the ICG solution (0.025 mg/ml) plus a drop of blood (Fig. 2). The basal values of heart rate and arterial blood pressure were recorded. For the inflation test, both balloons were inflated with approximately 15 ml of the ICG solution, and hemodynamic parameters were obtained after 1 and 3 min. The balloons were then deflated, and hemodynamic measurements were repeated twice. For the IVC injury test, using scissors, a 3- to 4-mm circumferential incision injury was created in IVC at the anterior wall. After creating the IVC incision, grade 0 hemorrhage (brisk steady bleeding) was confirmed, as Xie et al. previously described [6]. After a 5-s interval, the balloons were inflated for temporary hemostasis. If the bleeding was not well-controlled, balloons were partially deflated and moved so that the IVC injury was just above the balloon. In two of the five animals, we attempted to suture the injured IVC. Hemodynamic parameters, blood loss, and hemostatic time during IVC injury were intraoperatively recorded. Blood loss and hemostatic time are expressed as mean ± standard deviation. In this study, statistical analysis was not performed.

## Results

**Fig. 3:**
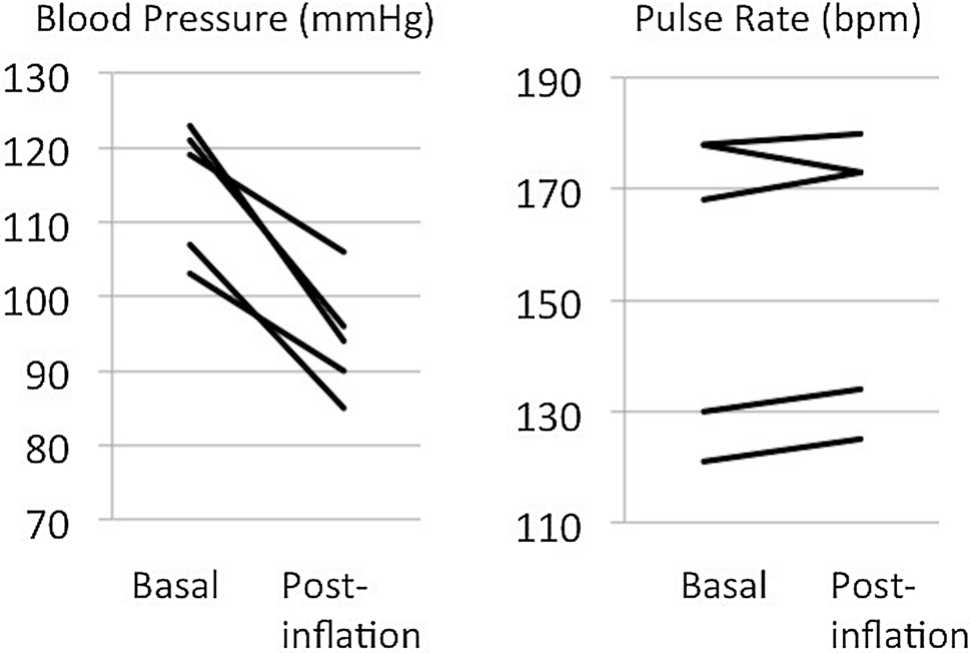
Effect of balloon inflation on hemodynamic status in five animals

**Fig. 4:**
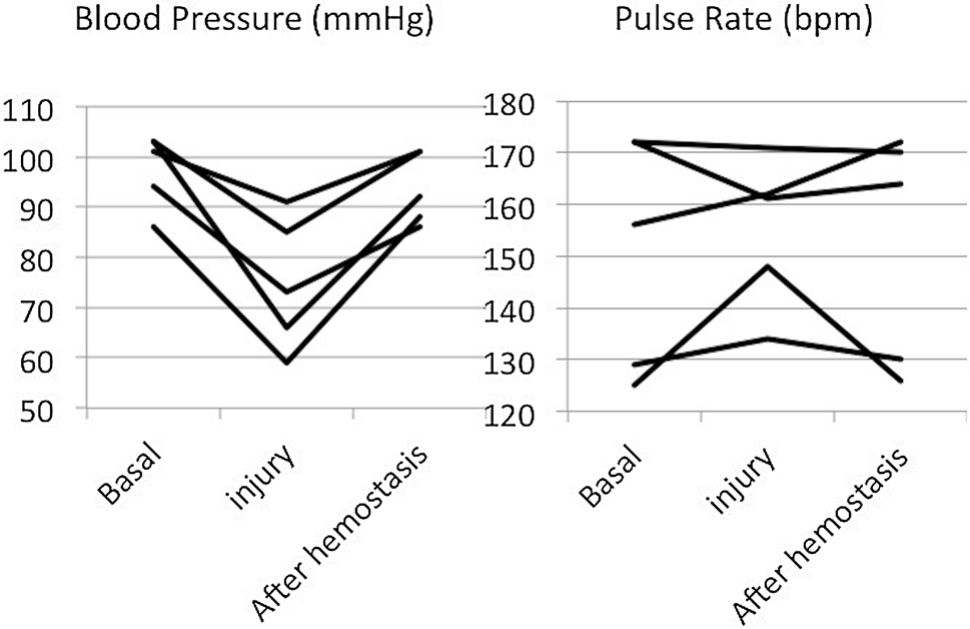
Hemodynamic change during IVC injury test in five animals

**Fig. 5:**
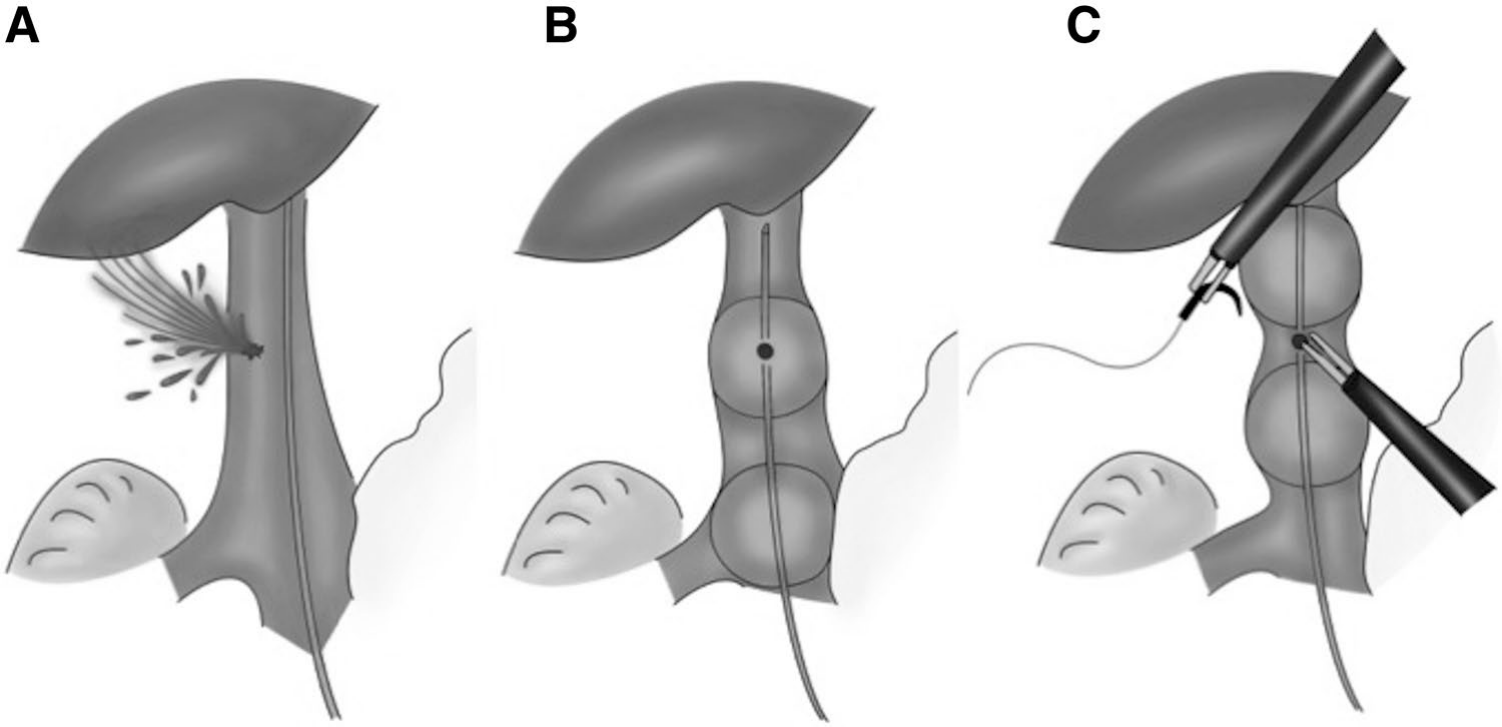
Picture demonstrating concept of intravenous balloon use. **A** IVC injury, **B** Temporary hemostasis by direct compression with inflated balloon, and **C** Repositioning of two dilated balloons facilitates suturing of the IVC injury

The mean arterial pressure was temporarily decreased by approximately 20 mmHg in all animals by IVC occlusion (Fig. 3). Clear observation of intravenous balloons was achieved using a near-infrared fluorescence camera, and accurate balloon placement was easily obtained (Fig. 2). During the IVC injury test, bleeding from the IVC injuries was successfully temporarily stopped with direct compression of a dilated balloon, with a mean time to hemostasis of 69 ± 44 s and blood loss of 32 ± 10 ml. During the compressions, the mean arterial pressure reduced by approximately 20 mmHg; however, it rapidly recovered to baseline following balloon deflation (Fig. 4). For prompt hemostasis, direct compression of the IVC injury by one of the two balloons was very effective. Subsequent placement of IVC injuries between two balloons made it possible to suture the injured IVC (Fig. 5).

## Discussion

As performing laparoscopic major hepatectomy continues to increase, the dissection of liver from IVC during laparoscopic surgery is also increasingly performed. IVC injuries, one of the most serious intraoperative complications, may easily occur. The mortality rate following an IVC injury reportedly ranges from 16 to 32%, despite aggressive treatment [7–9]. Surgeons should undertake gentle dissection around IVC to avoid iatrogenic injury and should be prepared to cope with massive bleeding. In this regard, our new device, the DB-CV catheter, is an invaluable aid for rapid, temporary hemostasis and has the potential to prevent massive hemorrhage from IVC.

To reduce the blood loss during laparoscopic hepatectomy, Zhu et al. attempted using balloon catheters to decrease the flow through the hepatic vein in an experimental animal model [10]. Although the device appeared to be an alternative tool for IVC injury, it was not verified for general use. Because the incidence of iatrogenic IVC injury during laparoscopic surgery is low (range 0.01–1.98%), routine insertion of a balloon catheter is not necessary. Moreover, once IVC injury accidentally occurs, surgeons do not have sufficient time to insert such a catheter to control vigorous bleeding. Promptness is the primary consideration in this situation. One of the novel features of our device is that the CV catheter is readily equipped with the balloons. Because low CV pressure anesthesia has become the standard anesthetic technique for hepatic resection, a CV catheter is routinely placed in the VC [11]. Should the catheter have a balloon function, it would facilitate rapid treatment of accidental IVC injury.

Another unique feature of our device is the double balloon. As described in Fig. 5, rapid and reliable hemostasis can be obtained with direct compression of the inflated balloon. Following temporary hemostasis, surgeons can choose either open conversion or laparoscopic suturing. In our experiments, we successfully attempted laparoscopic suturing in two of five animals, resulting in a bloodless field. To secure space for suturing, the two balloons were positioned on either side of the IVC injuries. Double balloons facilitate the laparoscopic suturing of IVC injury. Moreover, one balloon can compress the injury site even if the other balloon is unusable because of uncontrollable circumstances, such as rupture or blockage. To the best of our knowledge, a DB-CV catheter has not been previously reported.

The major concern with the device is hemodynamic change during complete occlusion of IVC. Temporary but complete occlusion of IVC has been reported in various clinical settings, including hepatic resection. Kato et al. conducted a randomized controlled trial that evaluated the effect of IVC clamping on blood loss during hepatic transection [12]. Although they failed to demonstrate any beneficial effects of IVC clamping, their data clearly showed that the effect of IVC clamping on systemic hemodynamics is minimal. Similar results were reported in the resection of renal tumors using a balloon to temporarily occlude VC [13]. The data from these previous reports are consistent with our results. In fact, reductions in arterial blood pressure of approximately 20 mmHg were observed in our experiments. Yang et al. explained the minor influence on systemic hemodynamics as being because of the precise positioning of the occlusion balloon just below the orifice of the hepatic veins. In addition, they concluded that the use of veno-venous bypass was deemed unnecessary during IVC occlusion. However, to further minimize the influence of IVC occlusion, equipping a central tube in the balloon to enable blood shunting may be of benefit, as demonstrated by Zhu et al. [10].

In Japan, the number of laparoscopic major hepatectomy procedures is likely to increase by virtue of national insurance coverage. Accordingly, opportunities for surgeons to inflict IVC injury will also increase. A new device, the DB-CV catheter, can rapidly and easily control severe IVC hemorrhage before a life-threatening situation arises. It is a useful contingency tool for surgeons during laparoscopic surgery and can help ensure patients’ safety.
